# Time-Related Alteration in Flow- (Shear Stress-) Mediated Remodeling in Resistance Arteries from Spontaneously Hypertensive Rats

**DOI:** 10.1155/2014/859793

**Published:** 2014-05-08

**Authors:** Odile Dumont, Gilles Kauffenstein, Anne-Laure Guihot, Nathalie C. Guérineau, Pierre Abraham, Laurent Loufrani, Daniel Henrion

**Affiliations:** ^1^CNRS UMR 6214, INSERM U1083, Université d'Angers, UFR de Médecine, rue Haute de Reculée, 49045 Angers, France; ^2^University Hospital Center (CHU) of Angers, rue Larrey, 49000 Angers, France

## Abstract

Hypertension is a major risk factor for cardiovascular disorders. As flow-mediated outward remodeling has a key role in postischemic revascularization, we investigated this remodeling in mesenteric resistance arteries of normotensive (WKY) and spontaneously hypertensive rats (SHRs) aged 3 to 9 months. Sequential ligation of mesenteric resistance arteries allowed modifying blood flow *in vivo*, thus exposing arteries to low, normal, or high flow. After 1, 3, 8, or 24 weeks, arteries were isolated for *in vitro* study. High flow (HF) induced outward hypertrophic remodeling in WKY rats after 1 week and persisted until 24 weeks without change in wall to lumen ratio. In SHRs, diameter increase was delayed, occurring only after 3 weeks. Nevertheless, it was reduced at 8 weeks and no longer significant after 24 weeks. In parallel, media cross-section area increased more with time in SHRs than in WKY rats and this was associated with increased contractility and oxidative stress with decreased NO-dependent relaxation. Low flow induced progressive inward remodeling until 24 weeks in both strains with excessive hypertrophy in SHRs. Thus, a chronic increase in flow induced transitory diameter expansion and long-lasting hypertrophy in SHRs. This could contribute to the higher susceptibility of hypertensive subjects to ischemic diseases.

## 1. Introduction

Arterial hypertension is a major public health problem concern worldwide. This insidious disease that causes few if any symptoms or warning signs is nevertheless an important risk factor for myocardial infarction, stroke, renal failure, and peripheral arterial disease. Chronic increase in blood pressure induces a structural vascular remodeling associated with endothelial dysfunction and increased vascular tone in resistance arteries [1, 2]. Hypertension-induced arterial remodeling is different along the vascular tree. Conduit arteries develop inward hypertrophic remodeling, whereas small arteries undergo inward eutrophic remodeling [1] in order to restore wall stress toward control level [3]. Nevertheless, hypertrophic remodeling and increased stiffness may affect resistance arteries in more severe forms of essential hypertension or in renovascular (secondary) hypertension [4] and this may have dramatic consequences on local perfusion pressure and blood supply to target organs.

Shear stress exerted by blood flow at the surface of vascular endothelium produces vasorelaxation and in the long term an outward arterial remodeling. Indeed, a chronic increase in blood flow induces arterial diameter expansion normalizing shear stress, while the associated compensatory hypertrophy normalizes wall strain [5, 6]. Such outward arterial remodeling occurs in response to regular physical exercise [7] during pregnancy [8] or in response to vasodilator treatments [9]. Moreover, flow-mediated outward remodeling is essential for collateral growth following ischemia [10]. Nevertheless, the mechanisms involved in dilation and hypertrophy may be dissociated. For example, in old healthy rats, diameter expansion does not occur, whereas hypertrophy remains in response to a chronic increase in flow [11]. On the other hand, in young healthy rats treated with an angiotensin II type 1 receptor blocker, diameter increases in response to a chronic increase in flow but in this case without vascular wall hypertrophy [12].

In previous studies, we have shown that nitric oxide (NO) is essential to mesenteric resistance arteries remodeling induced by a chronic increase in blood flow [13, 14]. The activity of endothelial NO synthase (eNOS) has been shown to increase in spontaneously hypertensive rats (SHR) with elevated NO [15] and cGMP [16] production. Nevertheless, reduced NO bioavailability has been established in hypertensive individuals, depending on the duration and severity of arterial hypertension [17]. Indeed, in SHRs, endothelium-derived constrictor factors (EDCFs) are produced, including angiotensin II, thromboxane A_2_, and endothelin-1 [18, 19]. The net result of EDCFs, reactive oxygen species (ROS) [15], and NO production by endothelial cells in SHR is an impaired endothelial function and vasodilatation compared to normotensive rats [17, 20].

High flow-mediated remodeling, when measured after 1 week, is reduced in young (10 weeks old) SHRs as compared to age-matched WKY rats [21]. Nevertheless, this is likely to be due to a different kinetic of remodeling in SHRs. In normotensive animals, a plateau of luminal expansion is reached after 1 week [22] while in SHRs, the reduced dilator response to flow [16, 20] might slow down the process. This may be the consequence of an elevated H_2_O_2_ level in SHRs arteries together with a high NO concentration that cannot be further elevated following chronically increased blood flow [15]. Although NO and ROS are both essential for high flow remodeling of large [23] and small arteries [24], oxidative stress is elevated in SHRs, so that the system could be unbalanced. Indeed, in the SHR, an antioxidant treatment restores high flow-mediated outward remodeling of the mesenteric artery [15]. Nevertheless, these studies were performed in 10-week-old SHRs, thus shortly after the onset of hypertension and most damage associated with high blood pressure occurs later. Finally, little is known about the evolution of remodeling with time in rats, especially in SHRs. In mature (10-month-old) [21] and old (24-month-old) rats [11], high flow-mediated remodeling is impaired. Although ROS production increases with age in normotensive rats, this phenomenon is exacerbated in hypertensive animals [25]. NAD(P)H-oxidase likely contributes to this age-related increase in ROS in hypertension [26].

In this study, we investigated the evolution and the nature of flow-mediated remodeling in the context of hypertension and aging. To address this issue, we conducted functional and biochemical studies on mesenteric resistance arteries chronically submitted to high blood flow for 1 week up to 6 months in 3-month-old WKY rats and SHRs to evaluate the parallel evolution of flow-mediated remodeling, arterial contractility, and endothelium-mediated relaxation.

## 2. Materials and Methods

### 2.1. Arterial Ligation in Rat Mesenteric Arteries

Forty-eight 10-week-old male WKY rats and 48 SHRs (Iffa-Credo, L'Arbresle, France) were anaesthetised (isoflurane, 2.5%) and pretreated with buprenorphine (Temgesic; 0.05 mg/kg, s.c.). A loop of intestine was then exposed and local mesenteric artery blood flow was surgically reduced as previously described [27, 28]. Briefly, from three adjacent first-order mesenteric arteries, second-order branches of the first and third arteries were ligated with 7-0 surgical silk threads. This creates high flow (HF) in the middle vessel, with low flow (LF) in the other two vessels. Control (normal flow, NF) vessels were distant first-order mesenteric arteries obtained from the same animal.

Rats were divided in 4 groups (*n* = 12 rats per group) and after 1, 3, 8, or 24 weeks, they were anesthetized for blood pressure measurement in the carotid artery [29] and for blood flow measurement in NF, HF, and LF arteries [22]. In short, blood flow was measured using a 1 mm ultrasonic flow probe and a TS420 transit-time perivascular flowmeter (Transonic System Inc.). Changes in blood flow were recorded for at least 10 min before the flow rate was averaged. Mesenteric arteries were then rapidly collected and used for arterial diameter measurement, histological analysis, or biochemical analysis.

The procedure followed in the care and euthanasia of animals was in accordance with the European Community Standards on the Care and Use of Laboratory Animals (Ministère de l'Agriculture, France, authorization number 6422). The protocol was approved by the regional ethical committee (Protocol CEEA PdL number 2008.10).

### 2.2. Arterial Diameter Measurement in Isolated Arteries

The mesenteric vascular bed was removed and kept in ice-cold physiological solution of the following composition (mM): 130, NaCl; 15, NaHCO_3_; 3.7, KCl; 1.2 KH_2_PO_4_; 1.2, MgSO_4_; 11, glucose; 1.6, CaCl_2_; and 5, N-2-hydroxy-ethylpiperazine-N-2ethylsonic acid, pH 7.4, PO_2_ 160 mmHg, and PCO_2_ 37 mmHg. Mesenteric arteries were dissected free of fat and connective tissue using a dissection microscope. From each HF, LF, and NF artery, one segment was quickly frozen in liquid N_2_, one was used to determine the pressure-diameter relationship, and one was used for a pharmacological study.

One segment was then cannulated at both ends in a video-monitored perfusion system (Living Systems Ins., Burlington, VT) as previously described [30]. Briefly, arteries were bathed and superfused with a Ca^2+^-free PSS containing EGTA (2 mmol/L) and sodium nitroprusside (SNP, 10 *μ*mol/L). Pressure was controlled by a servo-perfusion system (LSI, Burlington, VT) and increased by steps from 10 to 150 mmHg. Diameter changes were continuously measured and recorded (Biopac, MP100, La Jolla, CA).

At the end of each experiment, pressure was set at 75 mmHg, and the arteries were fixed in a 10% buffered formaldehyde solution, as previously described [31] and stored for histomorphometric analysis.

### 2.3. Histomorphometric Analyses

Sections (7 *μ*m thickness) were obtained from the fixed arterial segments and stained with orcein. External diameter, lumen diameter, and media thickness were determined after images acquisition (Olympus T100 microscope, Sony camera) and analyzed using the Histolab software (Microvision, Paris, France) for cross-sectional area (CSA) calculation as previously described [32].

### 2.4. Pharmacological Analysis

The last arterial segment (2 mm long) was mounted on a wire-myograph (DMT, Aarhus, DK) as previously described [33]. Briefly, 2 tungsten wires (25 *μ*m diameter) were inserted in the lumen of the arteries and connected to a force transducer and a micrometer, respectively. Arteries were bathed in the PSS described above [34]. A wall tension, equivalent to intra-arterial pressure, was applied [19] and vessels are allowed to stabilize for one hour. Artery contractility was assessed with phenylephrine (PE, 1 *μ*mol/L). Acetylcholine- (Ach 1 *μ*mol/L) induced relaxation was then obtained after phenylephrine-induced preconstriction (50% of maximal contraction) in the presence or in the absence of the NO synthesis blocker L-NAME (100 *μ*mol/L) or of superoxide dismutase (120 U/mL) plus catalase (80 U/mL) [31].

### 2.5. Tissue Extraction and Western Blot Analysis

Frozen arterial segments were pulverized in liquid nitrogen. The powders were resuspended in ice-cold lysis buffer: 150 mM NaCl, 1% IgepalCa630, 0,5% Na deoxycholate, 0,1% SDS, proteases inhibitors cocktail (Complete Protease Inhibitor Cocktail, Roche). Vessel extracts were incubated in this buffer on ice for 15 min and then centrifuged (14,000 rpm, 15 min, 4°C). The detergent soluble supernatant fractions were retained, and protein concentration in samples was equalized by using a Micro BCA Protein Assay Kit (Pierce) [11]. Proteins (15 *μ*g total protein from each sample) were separated by SDS-PAGE and transferred to nitrocellulose membranes. The membranes were incubated with the primary antibody (Transduction Laboratories for gp91phox, p67phox, and beta-actin 1 : 1000 in T-TBS, Santa Cruz Biotechnology for eNOS, 1 : 500 in TBS-T), washed (3 times for 15 min), and incubated with horseradish peroxidase-conjugated secondary antibody (Amersham) for 90 min at room temperature. The proteins were visualized using the ECL-Plus Chemiluminescence Kit (Amersham) and bands intensity was quantified by densitometry using Image J software. The results were normalized to beta-actin immunoreactivity [11].

## 3. Statistical Analysis

Results were expressed as means ± standard error (SEM). Significance of the differences between groups was determined by analysis of variance (two-way ANOVA for consecutive measurements followed by the Bonferroni *t*-test) to compare pressure-diameter curves in the different groups. In the other set of experiments, means were compared by unpaired Student's *t*-test. *P* values less than 0.05 were considered to be significant.

## 4. Results 

Mean arterial blood pressure was not significantly affected by the ligations in either strain. In the 4 studied groups (1, 3, 8, and 24 weeks of ligation) mean arterial pressure was 146 ± 6, 162 ± 7, 173 ± 9, and 170 ± 8 mmHg in SHRs and 92 ± 5, 95 ± 6, 94 ± 7, and 95 ± 7 mmHg in WKY rats (*n* = 12 per group).

Blood flow (Table 1), measured using a Transonic probe in NF arteries, ranged from 345 ± 33 to 402 ± 38 *μ*L/min in WKY rats (*n* = 6 per group, 1 to 24 weeks after surgery) and from 326 ± 38 to 350 ± 37 *μ*L/min in SHRs (*n* = 5 per group). In HF arteries blood flow ranged from 673 ± 52 to 724 ± 79 *μ*L/min in WKY rats (*n* = 5 or 6 per group) and from 654 ± 45 to 688 ± 70 *μ*L/min in SHRs (*n* = 5 per group, *P* > 0.05). In LF arteries, blood flow ranged from 110 ± 21 to 54 ± 16 *μ*L/min in WKY rats (*n* = 6 per group, *P* < 0.05) and from 93 ± 18 to 50 ± 14 *μ*L/min in SHRs (*n* = 5 per group, *P* < 0.05).

**Figure 1:**
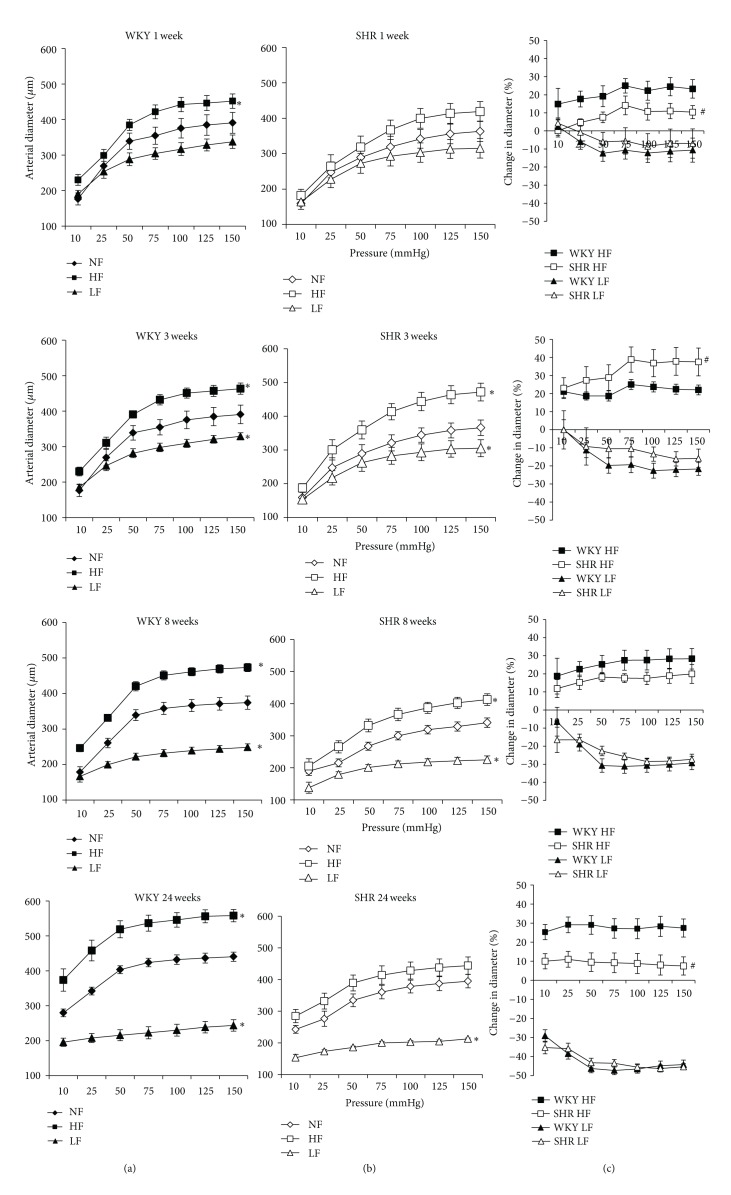
Pressure-diameter relationship determined in mesenteric resistance arteries isolated from normotensive rats (WKY: (a)) or spontaneously hypertensive rats (SHRs: (b)) submitted to arterial ligation for 1, 3, 8, or 24 weeks. Ligated arteries were designated as low-flow (LF) arteries. The artery located between two ligated vessels was designated as a high-flow (HF) artery. Other arteries had a normal flow (NF). Changes in diameter (C) due to remodeling in HF (positive values) and LF arteries (negative values) were calculated from the data shown in (a) and (b) and expressed as percentage change in diameter compared to NF vessels. Values representing mean ± SEM are shown (*n* = 12 rats per group). *P < 0.01, HF or LF versus NF arteries. ^#^ *P* < 0.05, SHRs versus WKY, rats within equivalent arteries (NF, LF, or HF).

In isolated mesenteric resistance arteries, stepwise increases in pressure induced a rise in diameter (Figures 1(a) and 1(b)). Arterial diameter was significantly higher in HF and lower in LF arteries than in NF vessels. In order to quantify remodeling, the diameter of HF and LF arteries was expressed as percentage of change compared to the NF artery (Figure 1(c)). In WKY rats, diameter in HF arteries was 20 to 28% higher than in NF vessels after 1 to 24 weeks (no significant difference between ages, Figure 1(a)). In SHRs, diameter rose by 8% in HF arteries after 1 week (not significantly different from NF vessels, Figure 1(c)). By contrast, diameter in HF vessels increased by 38% after 3 weeks (*P* < 0.01). Remodeling decreased then after 8 weeks (22%, *P* < 0.05 versus NF and *P* < 0.01 versus HF after 3 weeks) and after 24 weeks (9%, *P* < 0.01 versus 3 and 8 weeks).

Outward remodeling in HF arteries was significantly lower in SHRs than in WKY rats after 1 week and after 24 weeks, whereas it was higher after 3 weeks and equivalent after 8 weeks. Inward remodeling of LF arteries was equivalent in SHRs and in WKY rats and developed progressively with time (Figure 1(c)).

**Figure 2:**
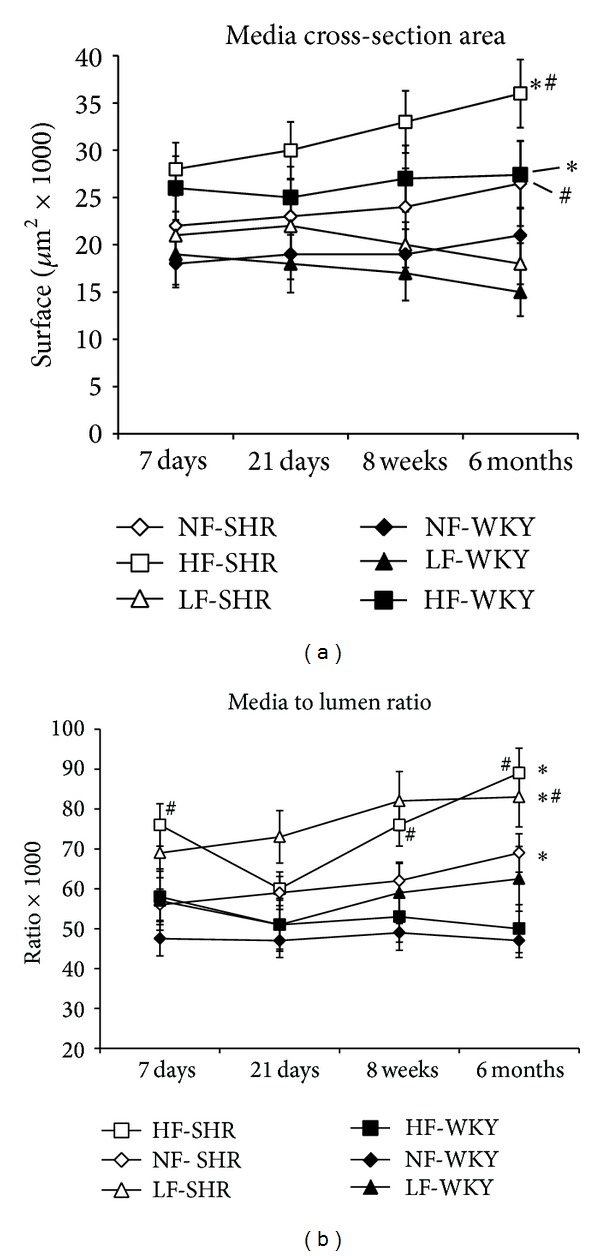
Media cross-section area (A) and media to lumen ratio calculated in mesenteric resistance arteries isolated from WKY rats and SHRs submitted to low flow (LF), high flow (HF), or normal flow (NF) for 1, 3, 8, or 24 weeks. Values represent mean ± SEM (*n* = 12 rats per group). *P < 0.01, HF or LF versus NF arteries. ^#^ *P* < 0.05, SHRs versus WKY rats, within equivalent arteries (NF, LF, or HF).

Media cross-section area (CSA) was higher in SHR compared to WKY rats arteries as previously described [1]. Chronic increase in blood flow (HF arteries) significantly augmented CSA in both SHR and WKY rats at 3, 8, and 24 weeks after ligation (Figure 2(a)). In LF arteries, CSA was not different when compared to NF vessels in both SHRs and WKY rats and there was no difference between the 2 strains. Media to lumen ratio, an index of hypertrophy, was significantly higher in SHR than in WKY in HF, LF, and NF arteries (Figure 2(b)). In WKY rats media to lumen ratio was not significantly different in HF and LF vessels than in NF arteries. In SHR, the ratio was higher in LF than in NF arteries. In HF arteries, the ratio was higher than in NF vessels in SHR after 1, 8, and 24 weeks but not after 3 weeks (Figure 2(b)).

**Figure 3:**
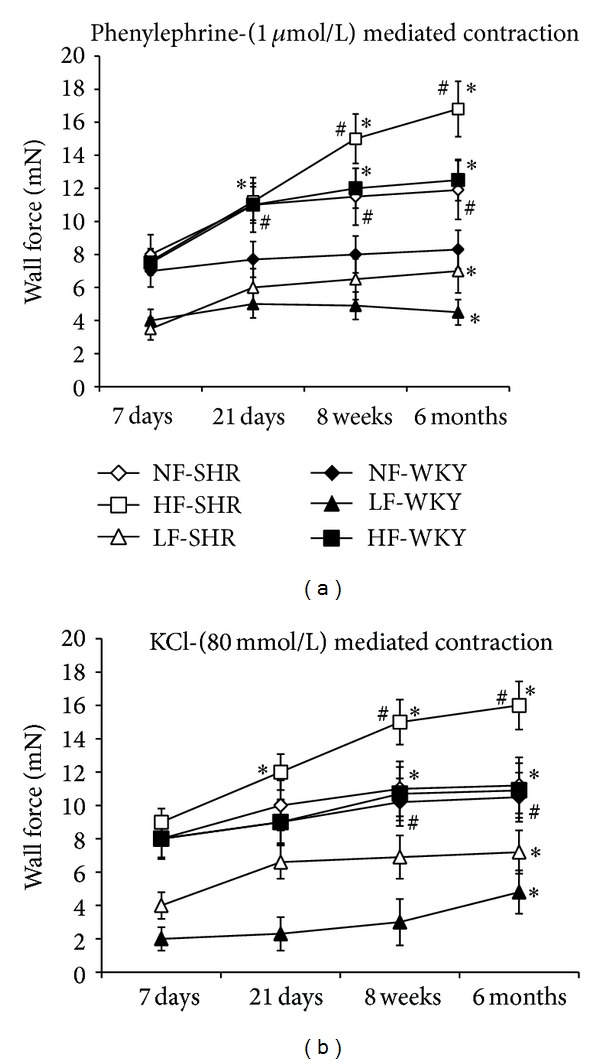
Phenylephrine- (1 *μ*mol/L, (a)) and KCl (80 mmol/L, (b)) evoked contraction measured in mesenteric resistance arteries isolated from WKY rats and SHRs submitted to low flow (LF), high flow (HF), or normal flow (NF) for 1, 3, 8, or 24 weeks. Values represent mean ± SEM (*n* = 12 rats per group). *P < 0.01, HF or LF versus NF arteries. ^#^ *P* < 0.05, SHRs versus WKY rats, within equivalent arteries (NF, LF, or HF).

Phenylephrine- (PE-) induced contraction (Figure 3(a)) was higher in HF and NF arteries from SHRs compared to WKY rats. No difference in contraction was observed in LF arteries between WKY rats and SHRs. In HF arteries, contraction to PE (1 *μ*mol/L) was higher than in NF vessels (significant after 8 and 24 weeks in SHRs and after 3, 8, and 24 weeks in WKY rats). Nevertheless, contractility rose progressively from 1 week to 24 weeks in HF arteries from SHRs, whereas the contraction was stable in HF arteries from WKY rat from 3 to 24 weeks. The contraction induced by depolarizing potassium concentration (KCl 80 mM) followed a similar pattern with the exception that it was higher in LF arteries in SHR compared to WKY rats (Figure 2(b)).

**Figure 4:**
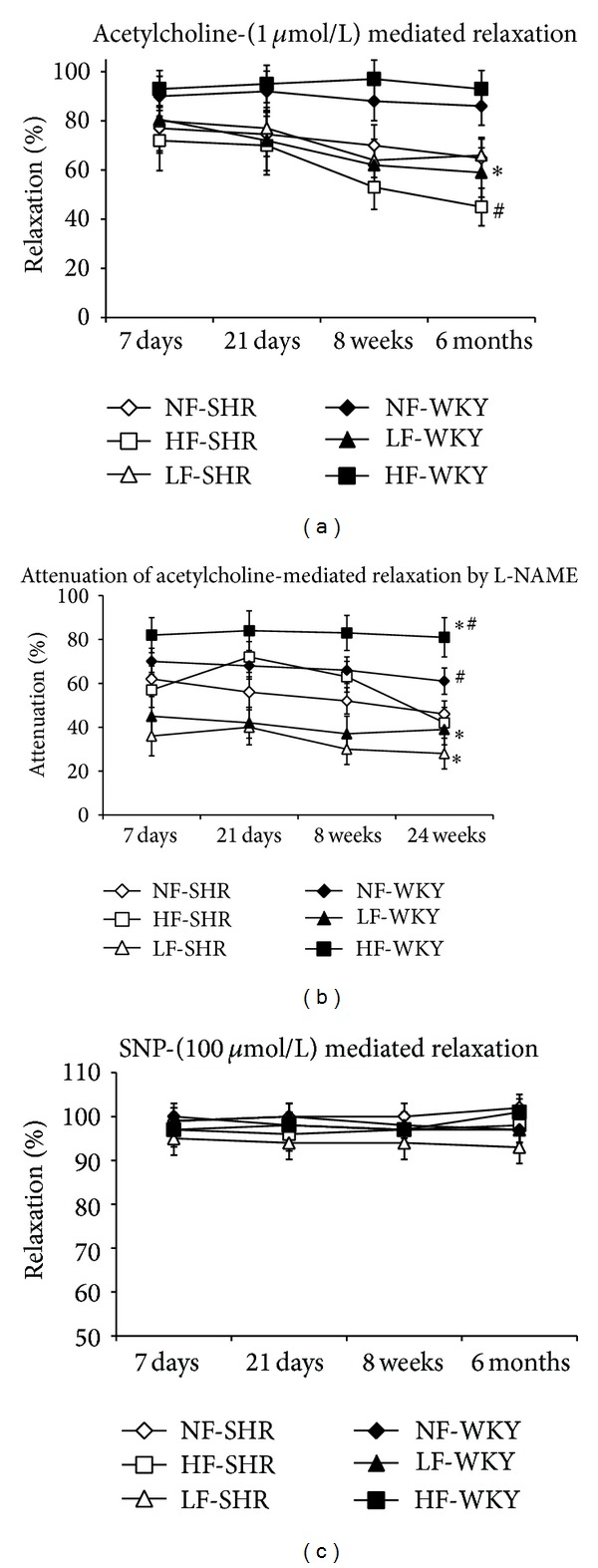
Acetylcholine- (1 *μ*mol/L) mediated relaxation (a), percentage attenuation of acetylcholine-mediated relaxation by L-NAME (b), and sodium nitroprusside- (SNP, 10 *μ*mol/L, (c)) mediated relaxation determined in mesenteric resistance arteries isolated from WKY rats and SHRs submitted to low flow (LF), high flow (HF), or normal flow (NF) for 1, 3, 8, or 24 weeks. Values represent mean ± SEM (*n* = 12 rats per group). *P < 0.01, HF or LF versus NF arteries. ^#^ *P* < 0.05, SHRs versus WKY rats, within equivalent arteries (NF, LF, or HF).

Endothelium-dependent relaxation in response to ACh (1 *μ*mol/L) was lower in HF and NF arteries from SHR than in equivalent arteries from WKY rats (Figure 4(a)). No difference in relaxation was observed in LF arteries between WKY rats and SHRs. In SHRs, ACh-mediated relaxation was reduced in HF compared to NF arteries. Attenuation of ACh-induced relaxation by L-NAME (Figure 4(b)) was lower in LF than in NF and HF arteries in both SHRs and WKY rats. No difference in sensitivity to L-NAME was observed between HF and NF arteries in both WKY rats and SHRs. Endothelium-independent relaxation induced by SNP was equivalent in all groups (Figure 4(c)).

**Figure 5:**
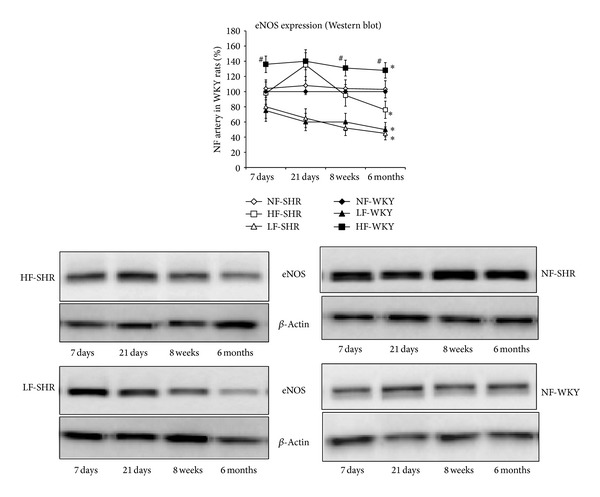
Endothelial NO synthase (eNOS) expression level was determined using western blot in mesenteric resistance arteries isolated from WKY rats and SHRs submitted to low flow (LF), high flow (HF), or normal flow (NF) for 1, 3, 8, or 24 weeks. Values represent mean ± SEM (*n* = 12 rats per group). *P < 0.01, HF or LF versus NF arteries. ^#^ *P* < 0.05, SHRs versus WKY rats, within equivalent arteries (NF, LF, or HF).

The expression level of eNOS was significantly higher in HF than in NF arteries in WKY rats, not in SHRs (Figure 5). It was therefore significantly lower in HF arteries from SHRs compared to HF vessels from WKY rats after 1, 8, and 24 weeks, not after 3 weeks. In LF arteries, eNOS expression level was significantly lower than in NF arteries, in both SHRs and WKY rats (no difference between the 2 strains).

**Figure 6:**
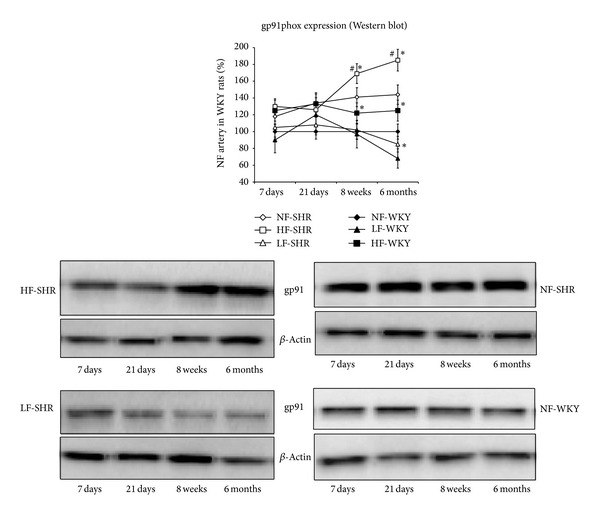
The NAD(P)H oxidase subunit gp91phox expression level was determined using western blot in mesenteric resistance arteries isolated from WKY rats and SHRs submitted to low flow (LF), high flow (HF), or normal flow (NF) for 1, 3, 8, or 24 weeks. Values represent mean ± SEM (*n* = 12 rats per group). *P < 0.01, HF or LF versus NF arteries. ^#^ *P* < 0.05, SHRs versus WKY rats, within equivalent arteries (NF, LF, or HF).

**Figure 7:**
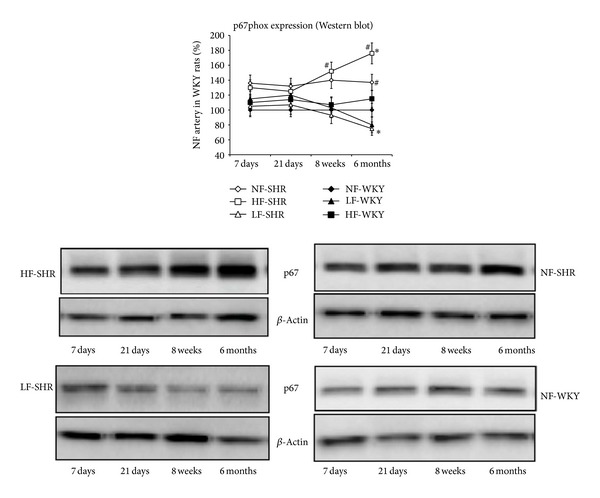
The NAD(P)H oxidase subunit p67phox expression level was determined using western blot in mesenteric resistance arteries isolated from WKY rats and SHRs submitted to low flow (LF), high flow (HF), or normal flow (NF) for 1, 3, 8, or 24 weeks. Values represent mean ± SEM (*n* = 12 rats per group). *P < 0.01, HF or LF versus NF arteries. ^#^ *P* < 0.05, SHRs versus WKY rats, within equivalent arteries (NF, LF, or HF).

The expression level of gp91phox (Figure 6) and p67phox (Figure 7) was significantly higher in HF than in NF arteries in WKY rats after 1, 3, 8, and 24 weeks and in SHRs after 8 and 24 weeks. The expression level of gp91phox and p67phox was also higher in SHRs than in WKY rats in NF arteries, but not in LF vessels. In HF arteries, gp91phox and p67phox expression level was higher in SHRs than in WKY rats only after 8 and 24 weeks.

**Figure 8:**
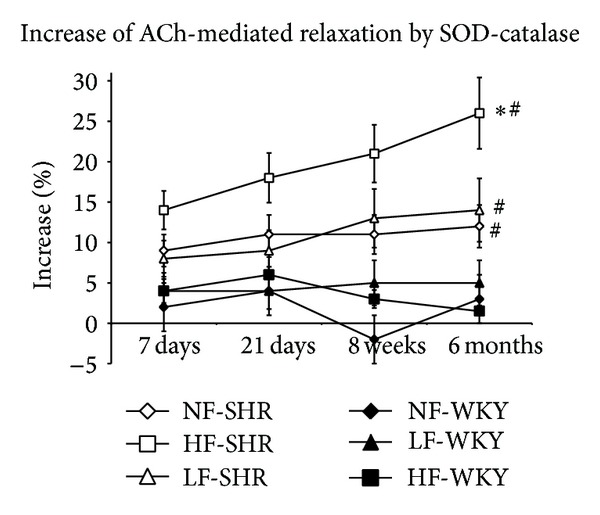
Percentage of increase of acetylcholine-mediated relaxation by superoxide dismutase (SOD) and catalase determined in mesenteric resistance arteries isolated from WKY rats and SHRs submitted to low flow (LF), high flow (HF), or normal flow (NF) for 1, 3, 8, or 24 weeks. Values represent mean ± SEM (*n* = 12 rats per group). *P < 0.01, HF or LF versus NF arteries. ^#^ *P* < 0.05, SHRs versus WKY rats, within equivalent arteries (NF, LF, or HF).

ACh-induced relaxation was increased by superoxide and catalase in arteries of SHRs but not in arteries of WKY rats (Figure 8). This effect was significantly greater in HF arteries than in NF and LF arteries in SHRs.

## 5. Discussion

Chronic increases and decreases in blood flow induce outward and inward arterial remodeling, respectively [27, 28]. These diameter changes allow the normalization of wall shear stress and are accompanied by a compensatory change in wall mass, which restores circumferential stress [27]. In order to investigate flow-mediated remodeling we used a model allowing the comparison of resistance arteries submitted* in vivo* to different blood flow levels in the same physiological conditions and in the same vascular bed for several days to several weeks.

Flow-mediated enlargement of preexisting vessels, or collaterals, constitutes a compensatory response to arterial narrowing or occlusion that allows adequate blood supply to distal ischemic tissues. The development of coronary collateral arteries allows reducing infarct size and increases survival in patients suffering coronary artery disease and was consequently proposed as a “valuable treatment strategy” in this pathology [35].

Collateral arterial growth is impaired in several physiological and pathological situations. Collateral growth is reduced in humans [36] and animals during aging [11, 21]. It is strongly impaired in diabetes, especially in the peripheral circulation such as the lower limb arteriolar network where improving collateral circulation is a key issue [37]. Finally, in hypertension a major risk factor for cardio- and neurovascular disorders is also associated with impaired flow-mediated remodeling in young SHRs and in patients suffering coronary artery disease [35]. However, the specific association of aging and hypertension has not been investigated and the duration of the increase in blood flow investigated in previous studies was limited to 1 week [15, 21].

In agreement with those previous studies [15, 38], high flow-mediated outward remodeling in SHRs was not significant 1 week after ligation of the adjacent arteries. Nevertheless, 3 weeks after ligation, a significant diameter expansion was observed in SHRs in association with improved NO-dependent relaxation and elevated eNOS expression. However, this did not persist and outward remodeling reversed progressively after 8 and 24 weeks. Thus, in SHR flow-induced diameter expansion was delayed compared to WKY and did not last over time. Several explanations could account for this result. First, the low kinetic of remodeling observed in SHRs could be due to oxidative stress, which is especially elevated in SHR arteries compared to WKY rats [15, 38]. Indeed, diameter expansion depends on the activation of matrix metalloproteinases by peroxynitrite which are formed from NO and O_2_^−^. Production of both NO and O_2_^−^ is activated by the chronic increase in flow as shown in the carotid [23] and in the mesenteric artery [22, 24, 39]. Nevertheless, an imbalanced equilibrium between NO and oxidative stress may alter remodeling. A previous work performed in young SHRs has shown that the chronic increase in flow is associated with elevated H_2_O_2_ level in the mesenteric artery together with a high NO concentration which cannot be further elevated despite the chronic increase in blood flow [15]. This is in agreement with the finding of the present study, as diameter expansion did not occur one week after increasing flow. In agreement, we found that arterial contractility, gp91phox, and p67phox levels as well as NO-dependent relaxation and eNOS level were not different in HF than in NF arteries in SHRs one week after ligation. By contrast, these parameters were all elevated in HF compared to NF arteries after 3 weeks. This observation is in favor of a lower kinetic of the process involved in high flow-mediated diameter expansion in the young SHRs.

In addition to the reduction in diameter expansion observed in the HF arteries after 8 weeks in SHRs, we found in these arteries a progressive increase in media cross-sectional area and contractility. This evolution of structure and vascular reactivity was observed neither in NF arteries in the SHRs nor in NF or HF vessels in WKY rats. The balance between NO and ROS is likely to be deleterious in HF arteries of SHRs as shown by our observations. The structural change observed in HF arteries in SHRs may be in favor of a net decrease in the biodisponibility of NO that has been shown to be a negative regulator of vascular smooth muscle proliferation in response to a remodeling stimulus [40].

Indeed, p67phox and gp91phox expression levels were higher in SHRs than in WKY rats. This may lead to excessive oxidative stress and consequently to hypertrophy and hypercontractility as previously described in various vascular territories in hypertension [41–43]. Our finding is also consistent with a previous study showing flow-mediated elevation of peroxide production in SHRs but not in WKY rats [15]. In human coronary arteries, flow-mediated dilation has also been associated with H_2_O_2_ production through activation of NADPH oxidase [44]. In the HF artery in SHRs, p67phox and gp91phox, hypertrophy and contractility increased continuously between 3 weeks and 6 months after ligation. The impaired outward remodeling observed in SHRs is in agreement with a previous study showing that antioxidant therapies reverse the impaired collateral arteries growth in the mesenteric circulation [38]. A possible explanation for the excessive oxidative stress and contractility found in SHR HF arteries is that the increased flow* per se* could induce this dysfunction. An acute increase in flow (shear stress) induces endothelium-mediated dilation, which is impaired in SHRs due to excessive vasoconstrictor agents (the so-called EDCFs) production $\left[\begin{array}{cc} 19 & 2000 \end{array}\right]$. After a chronic increase in blood flow, similar imbalance between vasodilator and vasoconstrictor agents may occur. This assumption is supported by our observations as well by previous studies cited above. As arterial diameter in the HF artery decreased after 3 weeks, shear stress was not normalized and consequently the stimulus (shear stress) remained elevated, possibly leading to a higher basal constrictor tone, which further reduced arterial diameter. This finding may help in understanding the bad outcome of ischemic disorders in hypertensive patients. Indeed, after occlusion of a large artery, outward arterial remodeling would not fully occur, thus reducing revascularization of the ischemic area. Our observation implies that this outward remodeling may not last over time and eventually deteriorate in hypertensive patients. Indeed, collateral growth has been shown to be impaired by hypertension [38, 45] as well as by other risk factors such as aging [36], metabolic syndrome [46, 47], and diabetes [48, 49].

Finally, in arteries exposed to a reduced flow, intraluminal diameter decreased continuously over the 6-month duration of the protocol without reaching a plateau and without difference between SHRs and WKY rats. Low flow-mediated inward remodeling is the consequence of unopposed constrictor tone as flow-mediated EDRF release is chronically reduced [12, 27, 50]. Consequently, diameter decreases without hypertrophy leading to eutrophic or hypotrophic inward remodeling as previously described [50–53]. This continuous diameter decrease is surprising and could not be attributed to the excessive contractility observed in SHRs as diameter followed a similar pattern in WKY rats. A possible explanation could be that collateral arterioles branching from the low LF artery also decrease in size due to a similar inward remodeling and progressively their density could also decrease and thus further reduce blood flow, thus resulting in a vicious circle. Nevertheless, this hypothesis remains speculative and thus it requires further investigation. Alternatively, the process of LF remodeling could involve a progressive and long-lasting decrease in endothelium- (NO) mediated relaxation, whereas contractility remains fully efficient. Thus this progressive unbalanced equilibrium between endothelium and smooth muscle would lead to a progressive reduction in arterial size.

To conclude we found a biphasic evolution of high flow-mediated outward remodeling of mesenteric resistance arteries in SHRs. Nevertheless, remodeling evolved rather negatively over time in SHRs with a progressive worsening of the arterial function and structure, whereas in WKY rats outward remodeling persisted over time with an improved endothelium-mediated relaxation. Such a reduced response to a chronic increase in blood flow could provide a possible explanation for the deleterious outcome of ischemic disorders in hypertensive patients.

## Figures and Tables

**Table 1:** Blood flow (µL/min), measured using a Transonic probe in NF, LF, and HF arteries of WKY rats and of SHRs.

Time after surgery	1 week	3 weeks	2 months	6 months
NF artery WKY rats	345 ± 33	354 ± 40	378 ± 46	402 ± 38
LF artery WKY rats	110 ± 21*	123 ± 23*	90 ± 21*	54 ± 16^∗$^
HF artery WKY rats	673 ± 52*	661 ± 64*	695 ± 70*	724 ± 79*

NF artery SHRs	326 ± 38	332 ± 41	367 ± 45	350 ± 37
LF artery SHRs	93 ± 18*	103 ± 24*	77 ± 18*	50 ± 14^∗$^
HF artery SHRs	654 ± 45*	598 ± 42*	645 ± 69*	688 ± 70*

Mean ± SEM is presented (*n* = 6 per group in the WKY rats and *n* = 5 per group in the SHRs).

*P < 0.05, HF or LF versus NF arteries.

^$^ *P* < 0.05, 3 weeks, 2 months, or 6 months versus 1 week.
